# Mental health struggles among newly qualified paramedics

**DOI:** 10.1038/s41598-025-28150-y

**Published:** 2025-11-22

**Authors:** Andrew Mosiane, Patricia McInerney, Simpiwe Sobuwa

**Affiliations:** 1 Department of Emergency Medical Care and Rescue, Faculty of Health Sciences, Durban University of Technology, Durban, 4001 South Africa; 2 Faculty of Health Sciences, Research Office, University of the Witwatersrand, Phillip V Tobias Health Sciences Building, 29 Princess of Wales Terrace, Parktown, Johannesburg, 2193 South Africa; 3 Department of Emergency Medical Sciences, Faculty of Health and Wellness Sciences, Cape Peninsula University of Technology, Symphony Way, Bellville, Cape Town, 7560 South Africa

**Keywords:** Emergency medical services, Mental health, Occupational stress, Paramedic, Psychological support, Transition shock, Health services, Occupational health

## Abstract

Healthcare workers face elevated risks of mental health problems, with paramedics particularly vulnerable due to trauma exposure. The transition from training to independent practice represents an especially challenging period, yet limited research exists on the mental health experiences of newly qualified paramedics in South Africa. Using an exploratory sequential mixed methods design with Critical Participatory Action Research methodology, we followed four newly qualified paramedics over 12 months. Data collection included six focus group discussions, eleven semi-structured interviews, and reflective journals. Participants included both graduate paramedics and 10 external Emergency Medical Services stakeholders, including educators and managers. Data were analysed using thematic analysis. Four key themes emerged: (1) Inadequate Resources and Support, leading to frustration and helplessness; (2) Pressure and Responsibility, causing mental strain from being the primary decision-maker; (3) Emotional Impact of Traumatic Events, triggering self-doubt and career questioning; and (4) Need for Mental Health Resources, emphasising the desire for specialised psychological support. The findings revealed that transition shock was exacerbated by insufficient support systems and the high-pressure nature of emergency care in South Africa. These findings highlight an urgent need for structured mental health support systems for newly qualified paramedics, including specialised psychological services and regular debriefing opportunities.

## Introduction

Healthcare workers are at increased risk for mental health problems, a risk exacerbated by the prolonged stressful conditions of the coronavirus pandemic^1–6^. While studies have documented elevated mental health risks among healthcare workers broadly, paramedics face unique psychological stressors due to their frontline role in emergency response, working in high-stress environments that demand quick decision-making and emotional resilience^7^. Exposure to trauma is particularly common, including being assaulted or threatened with weapons while on duty, being involved in motor vehicle accidents or managing severe emergencies in austere settings^8^. Research suggests that paramedics are most at risk of mental health problems among healthcare workers due to this elevated trauma exposure^9–11^.

The transition from training to independent practice further heightens this vulnerability. Studies describe a phenomenon of transition shock in early career paramedics, marked by increased rates of anxiety, depression, post-traumatic stress disorder (PTSD) and burnout compared with both experienced colleagues and the general population^10,12–16^. Systematic reviews by Berger et al.^9^ and Petrie et al.^17^, report pooled prevalence rates of 10% and 11%, far higher than the general population (1.3–3.5%)^9^. This underscores the significant psychological burden paramedics face.

The transition from a controlled training environment to the unpredictable realities of emergency medical service presents unique challenges. Paramedics are routinely exposed to traumatic events, life-threatening situations, and high-pressure decision-making scenarios that can have profound psychological impacts^8,10,13,15,18–20^. The discrepancy between theoretical training and practical field experiences, coupled with the emotional burden of patient outcomes, contributes to a complex landscape of stressors that can overwhelm new paramedics^7,16,21^.

Furthermore, organisational factors such as shift work, inadequate support systems, and the cultural stigma surrounding mental health in high-performance professions exacerbate the problem^10,22^. The cumulative effect of these stressors not only jeopardises the well-being of individual paramedics but also poses significant risks to patient care quality and the overall effectiveness of emergency medical services (EMS)^23^.

In South Africa, paramedics undergo extensive training, spending four years in higher education and completing approximately 1200 h of supervised clinical learning across diverse healthcare settings^24^. Despite this preparation, many newly qualified paramedics encounter significant psychological challenges, with burnout rates of 30%^25^, much higher than the 10–11% reported internationally^9,11^. This disparity could be attributed to the country’s high burden of interpersonal violence, to which paramedics are often the first responders^25,26^. These realities highlight the urgent need to understand and address the mental health crisis among newly qualified paramedics. By examining the psychological, social, and organisational stressors that shape the transition period, targeted strategies can be developed to support these essential professionals.

This article aims to highlight the challenges faced by newly qualified paramedics, with a particular focus on their mental health. Understanding and addressing this crisis is crucial not only for the well-being of paramedics but also for maintaining the integrity and effectiveness of emergency medical services.

## Methods

### Study design

The study was grounded in a Critical Theory paradigm, which emphasises agency, emancipation, and transformation in response to systemic inequities. Critical Theory informed both the research questions and the focus on actionable outcomes to empower graduate paramedics during their transition to practice.

Guided by this paradigm, we employed a Critical Participatory Action Research (CPAR) framework. CPAR was chosen because it aligns with Critical Theory in positioning participants as co-researchers, co-constructing solutions rather than serving solely as subjects of inquiry. This approach also ensured that findings were embedded in context, reflexive, and oriented toward practical change within the EMS system.

### EMS background/study setting

This study was conducted in KwaZulu-Natal (KZN), the province with the second largest population in South Africa. KZN has an estimated population of 11 065 240 people, representing approximately 19.7% of the national population^27^. The province is characterised by a high trauma burden and interpersonal violence, long transport distances and resource-constrained emergency medical services^28^. These contextual realities shaped both the transition experiences of graduate paramedics and the systematic barriers explored in this study.

In South Africa, three EMS qualification pathways exist:

1. a one-year Higher Certificate,
2. a two-year Diploma and
3. a four-year professional degree in emergency medical care.

All students must register with the Health Professions Council of South Africa to complete mandatory clinical placements throughout their studies. The HPCSA requires students to achieve specific clinical competencies before graduation. Graduates of the four-year professional degree qualify as Emergency Care Practitioners (ECPs)—the highest prehospital care registration category in South Africa^29,30^. Unlike in some countries, South Africa does not have an internship period post-graduation; instead, ECPs immediately enter independent practice as clinical leaders in their respective EMS organisations. Some EMS providers offer limited orientation programs, but there is no standardised transition framework, making the shift to independent practice highly variable.

### Participants and recruitment

Participants were recruited using purposive sampling from the 2021 Bachelor of Health Sciences in Emergency Medical Care (EMC) graduating class at a higher education institution in South Africa. Inclusion criteria were: (1) 2021 EMC graduates from a single institution in South Africa, (2) valid registration as ECPs, and (3) employed in clinical patient care within a South African public or private EMS organisation. There were 30 students initially registered in 2018, with 11 in their fourth year in 2021. However, only five students had met all the requirements of the programme. Of the five eligible graduates, four consented to participate. While this sample size appears small, it is justified by the longitudinal design^31,32^. Furthermore, small sample sizes are not unusual in qualitative research, as the research is contextual and influenced by the investigation being undertaken^31–33^. Data were collected through multiple touchpoints over 12 months, allowing rich, in-depth insights into the transition experience. Additional participants (see Table 1) (n = 10) were recruited via snowball sampling, representing public and private EMS educators from higher education institutions, public and private EMS operations, representatives from the National Department of Health and the HPCSA. These participants were initially recruited through the graduate paramedics, who recommended persons from the education and operations sectors who they thought would be able to contribute to the study, and then through the persons in these sectors who further identified others who could contribute to the study. These persons were contacted directly by the first author. Delphi panellists were identified by both the graduates and the external stakeholders as persons deemed highly knowledgeable, skilled and respected within the South African EMS setting. These experts had to represent both public and private EMS educators from the higher education institutions, public and private EMS operations, representatives from the National Department of Health and the HPCSA. The Delphi panellists were different participants from the external stakeholders and were contacted directly by the first researcher, unknown to each other. The Delphi study is covered in detail in a separate research paper.

**Table 1:** External EMS participants characteristics.

Participant	Position	Qualifications	Sex	Years in EMS
1	Private EMC HEI Educator	Master’s Degree	Female	15–20
2	Private EMC HEI Educator	Bachelor’s Degree	Male	15–20
3	Public EMC HEI Educator	Master’s Degree	Male	5–10
4	Public EMS Operations Manager	Bachelor’s Degree	Female	5–10
5	Private EMS Operations Manager	Bachelor’s Degree	Female	15–20
6	Public EMC HEI HOD	Master’s Degree	Male	> 20
7	Private EMS Operations Manager	Bachelor’s Degree	Female	5–10
8	Public EMC HEI HOD	PhD	Male	> 20
9	Public EMS Operations Manager	Bachelor’s Degree	Male	15–20
10	EMS Policy Maker	Bachelor’s Degree	Male	15–20

EMC, Emergency Medical Care; HEI, Higher Education Institution; HOD, Head of Department; EMS, Emergency Medical Services.

### Data collection

**Fig. 1:**
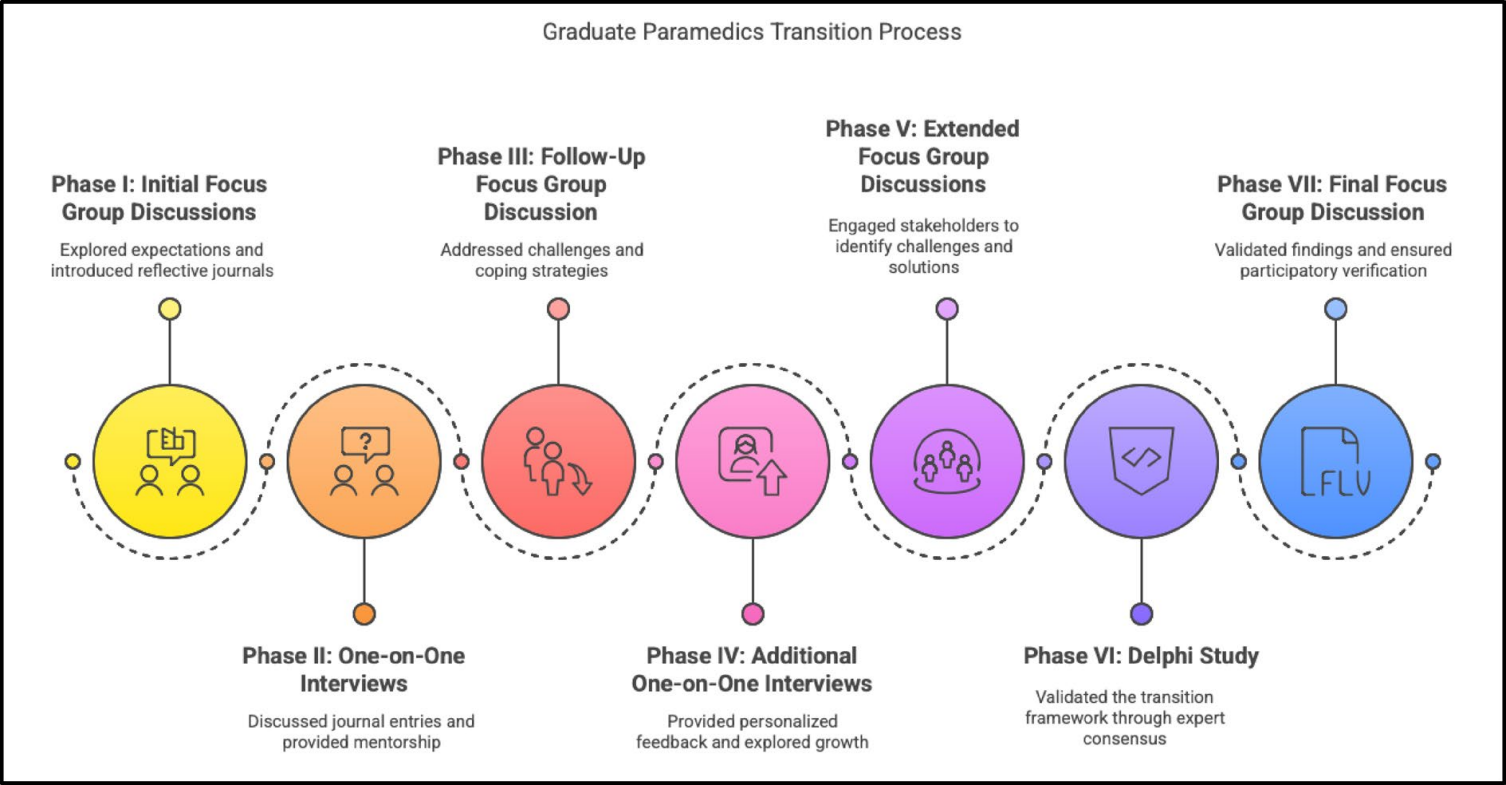
Structured seven-phase data collection process used in the study.

A structured seven-phase data collection process was implemented, designed to align with the CPAR methodology and to capture the evolving experiences of graduate paramedics over their first year of independent clinical practice (Fig. 1). Each phase combined different qualitative methods to ensure both depth and breadth of data, while allowing the participants to play an active role in shaping the research process.

### Phase I: Focus group discussion

A semi-structured FGD was conducted with the graduate paramedics at the inception of their independent practice. This discussion explored their expectations of the clinical transition and introduced the use of reflective journals as a tool for documenting significant clinical experiences.

### Phase II: One-on-one interviews

Three months into independent practice, semi-structured interviews (n = 4) were conducted with each graduate paramedic. These interviews centred on entries from their reflective journals, enabling the researcher to explore participants’ clinical experiences in depth while also providing guidance and feedback.

### Phase III: Focus group discussion

At the same time, a semi-structured FGD was conducted with the graduates. This discussion revisited the research problem, aims, and questions in light of their early clinical experiences. Consistent with the CPAR framework, this stage ensured that participants could confirm, refine, or amend the research problem, thus strengthening both the validity of the study and their sense of ownership in the research process^34^.

### Phase IV: One-on-one interviews

Five months into independent practice, a further series of semi-structured interviews (n = 4) was conducted with the graduates. These interviews again drew on reflective journal entries, which captured key moments in their clinical transition. This phase continued the iterative process of combining personal reflection with guided discussion and researcher feedback.

### Phase V: Focus group discussion with external stakeholders

Six months into practice, extended FGDs were conducted with external EMS stakeholders (n = 2), including educators, operations managers and policy representatives. Graduates had been invited to participate in this session, but none were willing to do so, given the mixed composition of the group. This phase allowed the perspectives of system stakeholders to be integrated with graduate experiences, extending the focus from individual trajectories to organisational and systemic contexts. A separate FGD was conducted with the graduate paramedics to propose a praxis to support clinical transition of graduates within the South African EMS milieu. They were subsequently given the opportunity to review and reflect on the praxis advanced by the external stakeholders addressing the same transition.

### Phase VI: Delphi study

At nine months, a Delphi process was undertaken with EMS experts to establish consensus on support mechanisms required to facilitate graduate transition. This represented the quantitative component of the exploratory sequential mixed methods design and provided an independent validation of the themes that had emerged in earlier phases. Three interviews were conducted with graduate paramedics to check in with them following their request for clinical support.

### Phase VII: Focus group discussion

Twelve months into independent practice, a final FGD was conducted with the graduate paramedics. During this session, the results of the Delphi study were presented and critically discussed. The graduates verified the relevance and applicability of the Delphi-validated praxis against their lived experiences, thereby reaffirming their central role as co-constructors of knowledge within the CPAR framework. This session also served to conclude the study collaboratively between the researcher and participants.

Across all phases, sessions were conducted via Microsoft Teams® due to geographical distance and COVID-19 restrictions. All FGDs and interviews were audio recorded with participant consent, transcribed verbatim, and returned to participants for member checking. Reflective journals, FGDs, and interviews were integrated to provide a rich longitudinal dataset that tracked both intra-individual change and cross-case patterns.

### Data analysis

Thematic analysis followed Braun and Clarke’s (2006) six-phase framework, supported by NVivo® Version 12 software for data organisation. A preliminary codebook was inductively developed by the first author after repeated reading of transcripts. This codebook was iteratively refined in collaboration with the co-authors to ensure conceptual clarity and consistency. Initial coding was conducted by the first author and co-coded by the other authors; any disagreements in coding were resolved through discussion until consensus was achieved. Member checking of transcripts further enhanced the accuracy and credibility of the analysis. Although formal intercoder reliability statistics were not calculated, the collaborative co-coding process strengthened consistency across the dataset. An inductive approach guided code and sub-theme development, while the final theme interpretation was informed by Critical Theory’s social factors framework. In practice, this meant situating participants’ accounts within the broader organisational and systemic conditions shaping their transition, including structural barriers, power dynamics, and inequities in support. This critical stance enabled the findings to move beyond description and highlight the underlying social determinants of graduate paramedics’ mental health challenges.

Data saturation was assessed throughout the longitudinal phases. By Phase IV, no substantially new codes were emerging from the interviews or reflective journals, and later phases primarily elaborated or deepened existing categories. The Delphi and final FGD confirmed and validated these established themes rather than generating new ones, indicating that longitudinal saturation had been achieved.

### Trustworthiness

Several strategies were employed to enhance methodological rigour. Prolonged engagement was achieved through the 12-month longitudinal design, which allowed repeated interaction with participants across different stages of their transition. Triangulation was built into the study through multiple data sources (focus groups, interviews, reflective journals), providing a richer and more nuanced understanding of experiences. Member checking of transcripts ensured that participants could verify the accuracy of the data and clarify their perspectives. Peer review and co-coding across the research team strengthened interpretive consistency, while reflexive practice allowed the first author to critically account for his positionality as an EMS educator and practitioner. Together, these measures enhanced credibility, dependability, and confirmability of the findings.

### Human ethics

Ethical approval was obtained from the Durban University of Technology Institutional Research Ethics Committee (IREC 293/21). All methods were carried out in accordance with relevant guidelines and regulations, and informed consent was obtained from all participants prior to participating in the study. Confidentiality was maintained through the use of pseudonyms and secure data storage. Psychological support services were available to participants if needed.

## Results

### Participant characteristics

Four graduate paramedics participated in the study. Pseudonyms are used to preserve confidentiality. Two participants (Tumi and Pat) entered practice without prior EMS experience, while Kgosi and Lerato both had previous EMS work experience. Following graduation, Tumi and Pat were employed in private EMS services, whereas Kgosi and Lerato continued working in private and public EMS organisations, respectively. None of the graduates had selected Emergency Medical Care (EMC) as their first choice of study when applying to university (see Table 2).

**Table 2:** Graduate paramedic characteristics.

Pseudonym	Prior EMS Experience	Employer Type Post-Graduation	EMC as First Study Choice
Tumi	None	Private EMS	No
Pat	None	Private EMS	No
Kgosi	Yes (private EMS)	Private EMS	No
Lerato	Yes (public EMS)	Public EMS	No

EMC, Emergency Medical Care; EMS, Emergency Medical Services

### Thematic findings

**Fig. 2:**
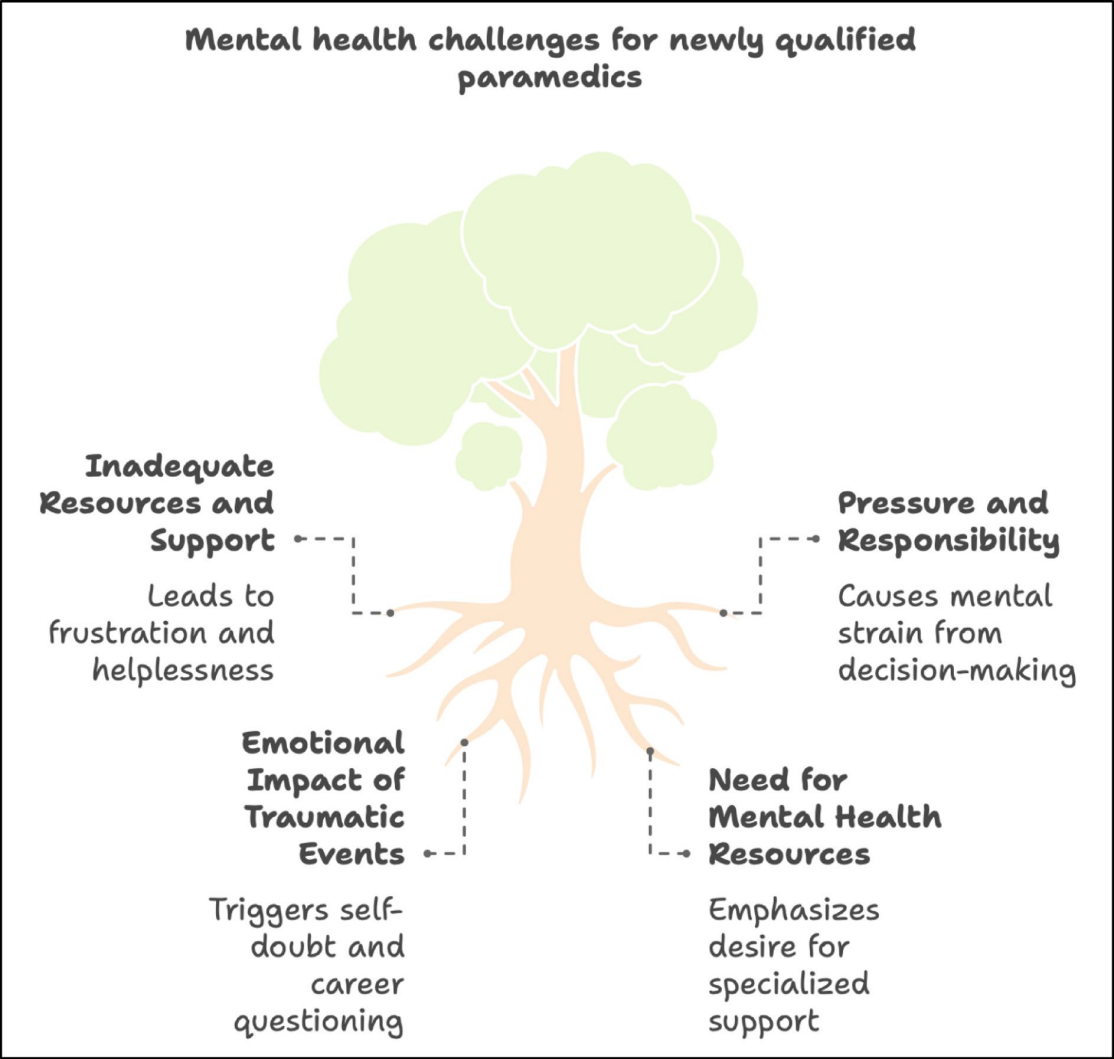
Conceptual representation of mental health challenges experienced by newly qualified paramedics.

The analysis generated four themes (see Table 3) that capture the mental health struggles of newly qualified paramedics during their transition to independent practice: inadequate resources and support, pressure and responsibility, emotional impact of traumatic events and need for mental health resources. Each theme is presented with supporting sub-themes and illustrative quotes to reflect participants’ lived experiences. A conceptual representation of these interrelated challenges and their impact on the mental health of newly qualified paramedics is presented in Fig. 2.

**Table 3:** Thematic matrix.

Theme	Sub-themes/codes	Illustrative quotes	Implications
Inadequate Resources & Support	Lack of equipment; lack of clinical support; poor orientation	“You kind of struggle to get the equipment you need to work…” – Lerato	Leads to frustration, feelings of being overwhelmed, compromised care
Pressure & Responsibility	Sole decision-maker; high patient expectations; lack of experience	“I was the senior on scene and my very first resuscitation as an independent practitioner. I felt overwhelmed…” – Pat	Heightens mental strain, anxiety, transition shock
Emotional Impact of Traumatic Events	Exposure to death/trauma; self-doubt; questioning career choice	“I thought maybe the job was not meant for me, I should have chosen another career.” – Tumi	Increases risk of PTSD, burnout, career attrition
Need for Mental Health Resources	Desire for counselling; peer support; structured debriefing	“At work we should also get someone who will sit, maybe a professional who we can talk to about our mental health.” – Pat	Highlights systemic gap; calls for structured psychological support

### Inadequate resources and support:

Lack of equipment, clinical support, and training contribute to feelings of frustration, disappointment, and being overwhelmed.

*“The resources in terms of the equipment and clinical support (consultant on call) may also not be fully supported in EMS systems especially in the government structures. I feel very disappointed in the system I am working under.”- Lerato.*

*“You kind of struggle to get the equipment you need to work but at the same time, you are expected to perform at your best to help patients.”* – Lerato.

*“The other thing that is experienced by new paramedics is that they don’t have the right equipment to perform their duties. They don’t have the equipment that is supposed to assist in clinical care. When they are out there alone, their patient treatment is not of the standard that they are supposed to be giving because there is no one assisting them and there is no appropriate equipment to use.” – EMS Operations Manager.*

### Pressure and responsibility:

The weight of being the “hope” for patients and the sole decision-maker in critical situations can lead to mental strain and anxiety.

*“I think mentally, it is something that is a bit straining to them, knowing that they are what we refer to as the hope of the EMS or the patients." EMC Educator*

*“Today I had to resuscitate a patient on the side of the road after being involved in a car accident. I was the senior on scene and my very first resuscitation as an independent practitioner. I felt overwhelmed by the scenario and under a lot of pressure as all decisions had to be taken by me.” – Pat.*

### Emotional impact of traumatic events:

Exposure to challenging and emotionally distressing cases can trigger feelings of self-doubt, fear, and even thoughts of leaving the profession.

*“An ET tube dislodgement on a head injured patient on my first day at work… I was feeling terrible after the case and I thought maybe the job was not meant for me, I should have chosen another career." Tumi.*

*“Today I had to do my first ‘alone’ prehospital intubation on a 14-year-old male patient. Following this difficult and challenging clinical case, I had to endure the battery and onslaught of the company due to my management of the patient. The criticism and onslaught of my management had me in a lot of emotional turmoil.” – Kgosi.*

### Need for mental health resources

Participants explicitly expressed the desire for accessible and specialised mental health support to address the emotional challenges of the job.

*“At work we should also get someone who will sit, maybe a professional who we can talk to about our mental health. Maybe when we get emotional calls, someone who can talk to us after we do such incidents and such cases” – Pat.*

*“Even though it’s difficult, but if we had to have an ECP that was also a qualified psychologist that would make a big difference. If we had weekly or monthly meets with them and you had to go through all your emotional experiences as well as you know do some critical thinking activities of some degree to develop a more thorough critical thinking process with regards to clinical decision making” – Kgosi.*

## Discussion

The transition from student to independent practitioner is a critical period for graduate paramedics. This study highlights significant challenges faced by newly qualified emergency care practitioners (ECPs) in South Africa, including inadequate resources, immense pressure and responsibility, emotional distress from traumatic cases, and the lack of mental health support. These findings align with global concerns regarding the preparedness and support of healthcare graduates entering independent practice^12,13^.

A recurring theme in this study was the lack of necessary equipment and clinical support. Participants reported frustration and disappointment due to insufficient resources, which compromised their ability to provide optimal patient care. The absence of adequate equipment and clinical guidance places additional strain on young paramedics, forcing them to operate below the expected standard. This mirrors findings in other low- and middle-income countries (LMICs), where inadequate infrastructure limits healthcare delivery^35^. To mitigate this, healthcare systems must prioritise resource allocation and implement structured mentorship programs to support new practitioners.

Graduate paramedics often find themselves as the primary decision-makers in life-threatening situations. The weight of being the “hope” for patients, coupled with limited practical experience, results in significant mental strain. Participants expressed anxiety over their sudden shift from student to senior decision-maker. Studies from high-income countries (HICs) confirm that this transition period is universally stressful, with a negative impact on competence, reinforcing the need for structured internship models^36,37^. This pressure can be seen as a consequence of the hierarchical structure of EMS, where new graduates are often placed in vulnerable positions with limited power to influence systemic change.

Exposure to critical and often distressing cases emerged as a major challenge for young paramedics, with research confirming that increased patient severity directly correlates with higher distress risk among paramedics^38^. Cases such as failed intubations, paediatric resuscitations, and high-pressure trauma scenarios triggered self-doubt and, in some instances, thoughts of leaving the profession. This emotional burden is especially pronounced among first-year healthcare practitioners, who often lack adequate coping mechanisms^12,36,37^. Within the South African context, this emotional burden may be exacerbated by high rates of interpersonal violence, violent crime and exposure to infectious diseases such as tuberculosis^39^. Furthermore, the EMS culture, often prioritising stoicism and resilience, may discourage new graduates from seeking help, perpetuating a cycle of emotional suffering^11,16,17,21^.

The culture within EMS and other high-performance or paramilitary professions often emphasises strength and emotional control, fostering a form of “toxic masculinity” that discourages vulnerability and makes seeking help seem like a sign of weakness^11,16–18,40^. The hierarchical, mission-focused nature of these organisations can also prioritise operational efficiency over the emotional well-being of its members, treating practitioners as a means to an end. Therefore, the lack of readily available mental health support is not just an oversight but a reflection of a deeply ingrained cultural ethos that has historically de-emphasised emotional health.

Participants explicitly called for accessible and specialised mental health support within their work environment, with specific suggestions including on-site psychological services, peer support programs, and structured debriefing following critical incidents. This aligns with the growing global recognition of mental health support systems as essential in EMS^38,41^. While these support systems may be increasingly common in high-income settings, their availability within South African EMS remains limited. The lack of readily available mental health support can be seen as a reflection of a system that prioritises productivity and cost-cutting over the well-being of its workforce.

### Strengths and limitations

This study benefited from the use of a CPAR approach, which empowered participants to actively shape the research process and ensured the findings remained grounded in their lived experiences. The longitudinal design, spanning 12 months, allowed repeated engagement with participants and generated rich insights into the evolving challenges faced by new paramedics. The inclusion of multiple stakeholder perspectives (graduates, educators, employers, and policymakers) further strengthened the comprehensiveness of the study.

Several limitations must, however, be acknowledged. The small sample size (n = 4 graduates) and the focus on a single institution restrict the generalisability of the findings. While purposive sampling was justified by the longitudinal design, the experiences of these participants may not reflect those of all new ECPs in South Africa. The use of self-reported data (interviews, focus groups, and reflective journals) also carries the risk of social desirability bias.

The positionality of the first author as an experienced paramedic and EMS educator may have influenced both participant disclosure and data interpretation. While this shared professional identity facilitated rapport and trust, it could also have shaped the way participants narrated their experiences, either through enhanced openness or moderation of responses. To address this, the first author engaged in ongoing reflexive journaling to acknowledge and monitor his influence on the research process. In addition, co-authors who were not directly involved in participants’ education undertook co-coding and critical peer review to mitigate potential bias. This reflexive transparency, combined with collaborative analysis, strengthened the methodological rigour of the study.

### Implications for practice

Similar to international best practices, a structured, supervised internship should be introduced for new paramedics. Government and EMS employers must ensure adequate equipment and clinical support systems. Psychological support, including professional counselling and structured debriefing sessions, should be integrated into EMS practice. Graduate paramedics should have access to regular case reviews, peer mentorship, and critical thinking workshops to enhance clinical confidence and competence. By addressing these key areas, EMS systems can improve the resilience, well-being, and clinical performance of graduate paramedics, ultimately enhancing patient outcomes and reducing burnout rates within the profession.

## Conclusion

The study highlights the significant mental health challenges faced by newly qualified paramedics in South Africa, including inadequate resources, immense pressure and responsibility, emotional distress from traumatic cases, and the lack of mental health support. The transition to independent practice exacerbates these challenges, leading to distress, self-doubt and potential burnout. The findings from this study emphasize the urgent need for structured support systems to facilitate the clinical transition of graduate paramedics. By prioritizing mental health support and creating a structured transition framework, EMS organizations and policymakers can foster a more resilient and well-prepared paramedic workforce, ultimately strengthening the healthcare system as a whole.

## Data Availability

The data is available from the first author, Andrew Mosiane ( [Andrew.mosiane@gmail.com], upon reasonable request.
